# La Grippe

**Published:** 1890-06

**Authors:** W. A. Yingling

**Affiliations:** Nonchalanta, Kansas


### LA GRIPPE.

W. A. YINGLING, M. D., NONCHALANTA, KANSAS.

La grippe has come, done its work, and gone. No results, as only pure Homœopathy was used in this entire community. The "regulars" have all gone; they went long before la grippe came.

Bell., Ipec., Causticum, Bry. were the principal remedies. In a few cases Eup.-per., Nux-v., and Bapt. Rückert, in his *Therapeutics*, says: "Causticum smelling, was employed by Hahnemann against influenza, and two hours afterward Camph." This was in the la grippe epidemic of that day. I verified this in three patients, but by pellets per mouth.

